# A Comprehensive Pan-Cancer Analysis Reveals GRB7 as a Potential Diagnostic and Prognostic Biomarker

**DOI:** 10.7759/cureus.74907

**Published:** 2024-12-01

**Authors:** Areig M Attaelmanan, Shyma Alzubair, Abdalla S Ahmmed, Ahmed Abdelgalil, Fatima Ali, Amira S Khalafalla, Gamila A Attaelmanan, Mohamed Alfaki

**Affiliations:** 1 Biotechnology, Newcastle University, Khartoum, SDN; 2 Medical Laboratory Science/Histopathology and Cytology, Sudan University of Science and Technology, Khartoum, SDN; 3 College of Medicine, University of Sharjah, Sharjah, ARE; 4 Molecular Biology, Institute of Post Graduate Medical Education and Research, Khartoum, SDN; 5 Hematology, University of Khartoum, Khartoum, SDN; 6 Clinical Chemistry, Faculty of Medicine, University of Gezira, Wad Madani, SDN; 7 Medical Laboratory Sciences/Hematology, Al Neelain University, Khartoum, SDN; 8 Genetics/Bioinformatics, Sidra Medicine, Ar-Rayyan, QAT

**Keywords:** bioinformatics analysis, diagnostic biomarker, grb7, immune infiltration, kich, kirc, paad, pan-cancer, prognostic biomarker

## Abstract

Background and aim: Growth factor receptor-bound protein 7 (GRB7) belongs to a group of adaptor proteins characterized by their conserved multidomain structure. These proteins are involved in cellular signaling pathways that regulate cell growth, proliferation, and differentiation. Alterations in GRB7 expression have been linked to multiple human cancers. However, its role as a diagnostic and prognostic marker remains underexplored. This study aimed to assess the diagnostic and prognostic relevance of GRB7 in a comprehensive pan-cancer analysis.

Materials and methods: GRB7 expression across different cancers was evaluated using the Tumor Immune Estimation Resource (TIMER), Gene Expression Profiling Interactive Analysis (GEPIA), and the University of Alabama at Birmingham Cancer Data Analysis Portal (UALCAN). The correlation of GRB7 expression with various clinicopathological parameters was assessed by the UALCAN database. Additionally, the Human Protein Atlas (HPA) (https://www.proteinatlas.org/) was used to illustrate the histology of kidney cancer tissues. The correlation between GRB7 expression and prognosis was explored using the Kaplan-Meier plotter, GEPIA, and UALCAN databases. The TIMER database was used to explore the connection between GRB7 expression in tumor tissues and the infiltration of immune cells. Moreover, genetic alterations of the GRB7 gene were detected by the cBioPortal database. Results were validated by the GEO2R database.

Results: GRB7 expression was significantly upregulated in bladder urothelial carcinoma (BLCA), cervical squamous cell carcinoma (CESC), cholangiocarcinoma (CHOL), colon adenocarcinoma (COAD), rectum adenocarcinoma (READ), thyroid carcinoma (THCA), and uterine carcinosarcoma (UCEC). Conversely, it was downregulated in kidney chromophobe (KICH) and kidney renal clear cell carcinoma (KIRC) compared to normal tissues (p<0.001). Further analysis confirmed that GRB7 expression in KICH and KIRC was significantly downregulated across various clinicopathological parameters including stage 3 and stage 4 compared to stage 1. It was also significantly downregulated in 61-80 years compared to 41-60 years patients, as confirmed by the immunohistochemistry of kidney tissues. Prognostic analysis revealed that high GRB7 expression was linked to a better prognosis in KIRC and a poorer prognosis in pancreatic adenocarcinoma (PAAD) patients. In KICH, GRB7 expression showed a significant positive correlation with immune infiltration of B cells, CD8+ T cells, and macrophages. In KIRC, GRB7 was positively correlated with immune infiltration of B cells and CD4+ cells. However, in PAAD it was negatively correlated with immune infiltration of macrophages. These findings were validated by gene expression profiling from the Gene Expression Omnibus (GEO) database, confirming a significant GRB7 downregulation in KICH and KIRC and an upregulation in PAAD compared to normal samples.

Conclusion: GRB7 shows potential as a biomarker in both diagnosing and predicting outcomes for various cancers. It may serve as a diagnostic marker for KICH, a prognostic marker for PAAD, and both a diagnostic and prognostic marker for KIRC, making GRB7 a target for future research and therapeutic approaches in oncology.

## Introduction

Global cancer data show that cancer is a major global health burden. In 2022, there were over 10 million cancer-related fatalities and roughly 20 million new instances of the disease. According to expectations, there will be 35 million new instances of cancer by 2050, a 77% rise from 2022 [1].

With the rising incidence and mortality of cancer, understanding its onset, progression, and therapeutic strategies has become crucial. The development of new drugs targeting proteins within essential signaling pathways has accelerated due to advances in genomic research. These targeted inhibitors have demonstrated better efficacy and fewer side effects compared to traditional treatments. Among the promising targets are adaptor proteins, specifically those containing Src-homology 2 (SH2) domains [2]. Growth factor receptor-bound protein 7 (GRB7) is one such adaptor protein, part of a family that also includes GRB10 and GRB14. This family shares a conserved multidomain structure, consisting of a proline-rich region at the N-terminus, a pleckstrin homology domain, a Ras-associated domain, and a Src-homology 2 (SH2) domain [3]. Research has demonstrated that these GRB7 domains are an essential component of cellular signaling pathways and have a major impact on controlling cell growth, division, and proliferation [4]. GRB7 has been linked to growth factor receptors like EGFR and HER2, highlighting its involvement in a range of cellular processes [5]. It influences the proliferation, survival, and metastasis of cancer cells by interacting with the ErbB family of receptors [6]. GRB7 controls stress reactions and apoptotic pathways to promote cell survival. Because it knocks down the amounts of BCL-2 family proteins, it increases apoptosis [4,7]. GRB7 is associated with anchorage-independent growth and cell cycle progression in terms of proliferation, particularly in malignancies that overexpress ErbB23. High GRB7 expression increases the capacity for cancer cells to migrate, invade, and spread via interacting with different kinases and phospholipids [4]. GRB7 is the focus of much research in the domains of molecular biology and cancer biology due to its many roles. The GRB7 gene has been found to be dysregulated in various human cancers, indicating its potential as both a biomarker and a therapeutic target [7,8]. However, despite these findings, the functional role of GRB7 in cancer remains largely unexplored, especially in the context of its potential importance for molecular-targeted therapies in cancer treatment. Further analysis of the role of GRB7 in cancer could lead to the development of new therapeutic approaches. This work sought to explore the potential of GRB7 in cancer diagnosis and prognosis by performing a thorough pan-cancer investigation using several bioinformatics methods.

## Materials and methods

Gene expression analysis

GRB7 expression across different cancers was evaluated using the Tumor Immune Estimation Resource (TIMER) 2.0 (http://timer.cistrome.org/) database. It is an analysis network for tumor immune cell infiltration and can also perform differential gene expression analysis between normal and tumor tissues [9]. The GRB7 gene's differential expression across 32 types of tumors obtained from The Cancer Genome Atlas (TCGA) was analyzed using the TIMER database, and the Wilcoxon test was applied to determine the statistical significance of differential expression. Moreover, The Gene Expression Profiling Interactive Analysis (GEPIA) (http://gepia.cancer-pku.cn/) database was used. The GEPIA database integrates data from TCGA and Genotype-Tissue Expression (GTEx). The analysis of the GRB7 gene's differential expression across 33 types of tumors was performed using a cutoff of 0.05 for the p-value and 1.0 for the |log_2_(FC)| cutoff [10]. Further analysis was conducted using the UALCAN database (https://ualcan.path.uab.edu/) to assess the differential GRB7 gene expression in normal and cancer tissues and also to determine GRB7 expression at the protein level across various tumor types, leveraging data from the Clinical Proteomic Tumor Analysis Consortium (CPTAC) datasets (http://ualcan.path.uab.edu/analysis-prot.html), and to explore the relationship between GRB7 expression and clinical pathological parameters of cancer [11]. Moreover, the Human Protein Atlas (HPA) (https://www.proteinatlas.org/) was employed to illustrate the histological differences in cancer tissues [12].

Prognostic value of GRB7 in cancers

The prognostic value of the GRB7 gene in various cancers was evaluated using the Kaplan-Meier plotter database (https://kmplot.com/analysis) [13] and the GEPIA2 database (http://gepia.cancer-pku.cn/) [10]. The survival analysis module in GEPIA assessed overall survival (OS) and disease-free survival (DFS) based on GRB7 expression in patients with different tumor types from the TCGA database [4]. Furthermore, the relationship between GRB7 expression and both overall survival and relapse-free survival (RFS) across 21 cancers was derived from the Kaplan-Meier plotter. Hazard ratios (HRs) with 95% confidence intervals (CIs) were computed, and log-rank p-values were used to assess statistical significance. A p-value <0.05 was considered statistically significant for prognostic outcomes. Furthermore, the overall survival was examined using the UALCAN database (http://ualcan.path.uab/index.hrml) [11].

Immune cell infiltration analysis

TIMER (https://cistrome.shinyapps.io/timer/) is an online website applied to characterize immune infiltration patterns across various cancer types [9]. This study utilized the TIMER tool to explore the correlation between GRB7 expression and immune cell infiltration. The analysis focused on estimating the abundance of six types of immune cells - B cells, CD4+ T cells, CD8+ T cells, neutrophils, macrophages, and dendritic cells across various cancer types.

Genetic alteration analysis

The cBioPortal database (https://www.cbioportal.org/) was used to examine genetic alterations of the GRB7 gene across pan-cancer TCGA datasets [14]. By selecting the "TCGA Pan-Cancer Atlas Studies" and entering the "GRB7" gene through the query function, the search yielded a total of 10,967 samples from 32 studies.

Validation

Regarding data validation, publicly available datasets from the National Center for Biotechnology Information (NCBI) (https://www.ncbi.nlm.nih.gov/) were utilized [15]. The GEO2R tool (https://www.ncbi.nlm.nih.gov/geo/geo2r/) was applied to compare different sample groups for identifying differentially expressed genes. The significance of GRB7 in kidney chromophobe (KICH), kidney renal clear cell carcinoma (KIRC), and pancreatic adenocarcinoma (PAAD) was confirmed using several GEO datasets, including GSE11024, GSE53757, and GSE16515. Differential expression was visualized using volcano plots from the bioinformatics.com.cn platform [16]. Statistical significance was determined based on the criteria p-value <0.05, adjusted p-value <0.05, and |log_2_(FC)| >1 for detecting differentially expressed genes.

## Results

GRB7 gene expression analysis

**Figure 1:**
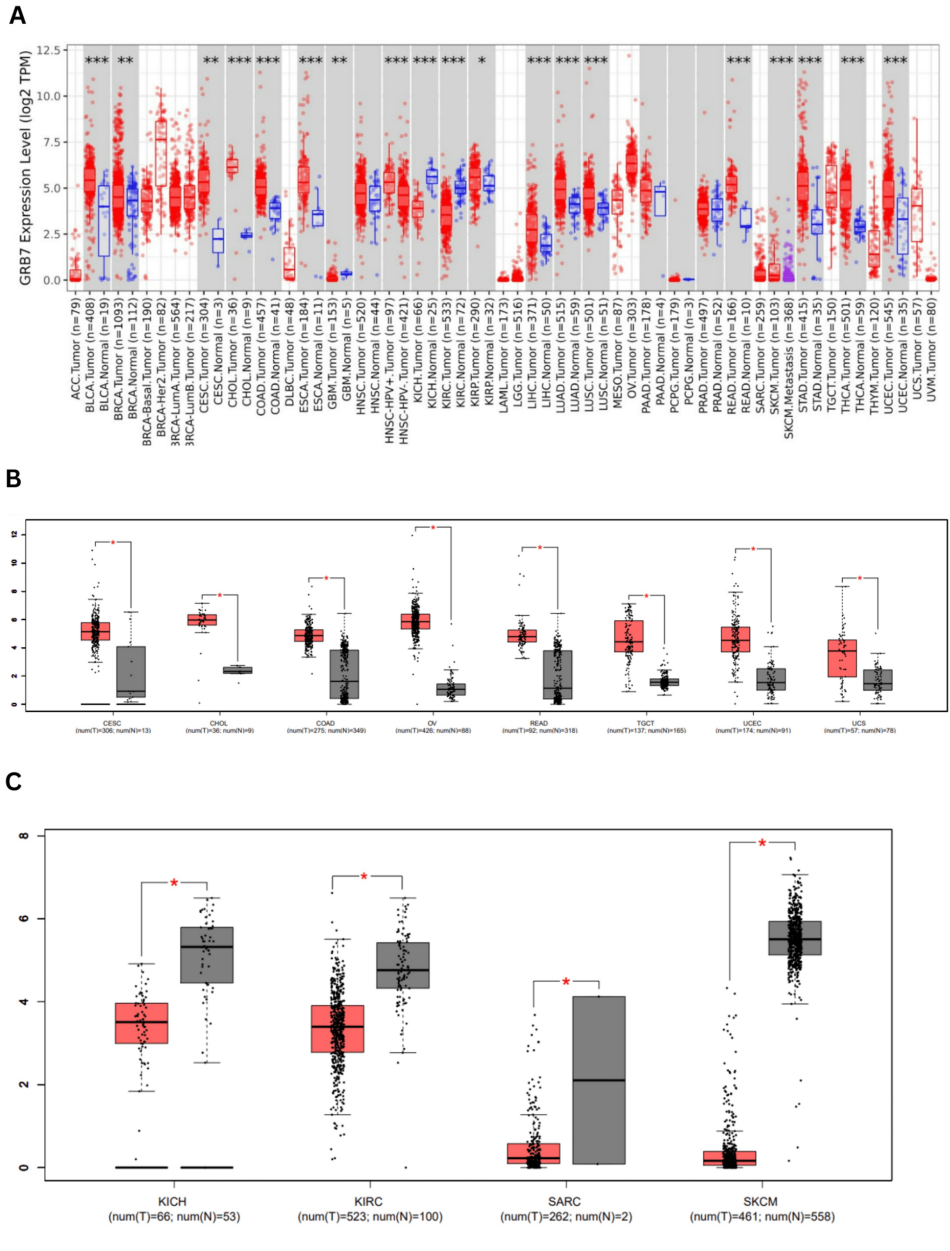
Pan-cancer analysis of GRB7 expression levels using TIMER and GEPIA. (A) GRB7 expression levels in various tumors using the TIMER2.0 database. (B) Cancers with significantly upregulated GRB7 expression using the GEPIA database. (C) Cancers with significantly downregulated GRB7 expression using the GEPIA database. The red columns represent the tumor tissues, and the blue and black represent the normal tissues, while the stars indicate the differential significance between the tumor and normal samples. *P<0.05 was considered significant. **P<0.01 was considered significant. ***P<0.001 was considered significant. GRB7: growth factor receptor-bound protein 7; TIMER: Tumor Immune Estimation Resource; GEPIA: Gene Expression Profiling Interactive Analysis; ACC: adrenocortical carcinoma; BLCA: bladder urothelial carcinoma; BRCA: breast invasive carcinoma; HER2: human epidermal growth factor receptor 2; LumA: luminal A; LumB: luminal B; CESC: cervical squamous cell carcinoma and endocervical adenocarcinoma; CHOL: cholangiocarcinoma; COAD: colon adenocarcinoma; DLBC: lymphoid neoplasm diffuse large B-cell lymphoma; ESCA: esophageal carcinoma; GBM: glioblastoma multiforme; HNSC: head and neck squamous cell carcinoma; HPV+: human papillomavirus positive; HPV-: human papillomavirus negative; KICH: kidney chromophobe; KIRC: kidney renal clear cell carcinoma; KIRP: kidney renal papillary cell carcinoma; LAML: acute myeloid leukemia; LGG: brain lower grade glioma; LIHC: liver hepatocellular carcinoma; LUAD: lung adenocarcinoma; LUSC: lung squamous cell carcinoma; MESO: mesothelioma; OV: ovarian serous cystadenocarcinoma; PAAD: pancreatic adenocarcinoma; PCPG: pheochromocytoma and paraganglioma; PRAD: prostate adenocarcinoma; READ: rectum adenocarcinoma; SARC: sarcoma; SKCM: skin cutaneous melanoma; STAD: stomach adenocarcinoma; TGCT: testicular germ cell tumors; THCA: thyroid carcinoma; THYM: thymoma; UCEC: uterine corpus endometrial carcinoma; UCS: uterine carcinosarcoma; UVM: uveal melanoma

GRB7 gene expression was assessed in cancerous and normal tissues using TIMER 2.0. The analysis revealed that GRB7 expression was significantly elevated across multiple cancer types, including bladder urothelial carcinoma (BLCA), cholangiocarcinoma (CHOL), colon adenocarcinoma (COAD), esophageal carcinoma (ESCA), liver hepatocellular carcinoma (LIHC), lung adenocarcinoma (LUAD), lung squamous cell carcinoma (LUSC), rectum adenocarcinoma (READ), stomach adenocarcinoma (STAD), thyroid carcinoma (THCA), uterine corpus endometrial carcinoma (UCEC) (p<0.001), breast invasive carcinoma (BRCA), cervical squamous cell carcinoma (CESC) (p<0.01), and kidney renal papillary cell carcinoma (KIRP) (p<0.05). In contrast, the expression of GRB7 was significantly reduced in glioblastoma multiforme (GBM) (p<0.01), KICH, and KIRC (p<0.001), as shown in Figure 1A. Additional analysis performed using the GEPIA database showed that GRB7 expression was significantly elevated in BLCA, CESC, CHOL, COAD, OV, READ, testicular germ cell tumors (TGCT), THCA, thymoma (THYM), UCES, and uterine carcinosarcoma (UCS) (Figure 1B). On the other hand, in KICH, KIRC, acute myeloid leukemia (LAML), skin cutaneous melanoma (SKCM), and sarcoma (SARC) the expression levels were lower compared to normal tissues, as shown in Figure 1C.

**Figure 2:**
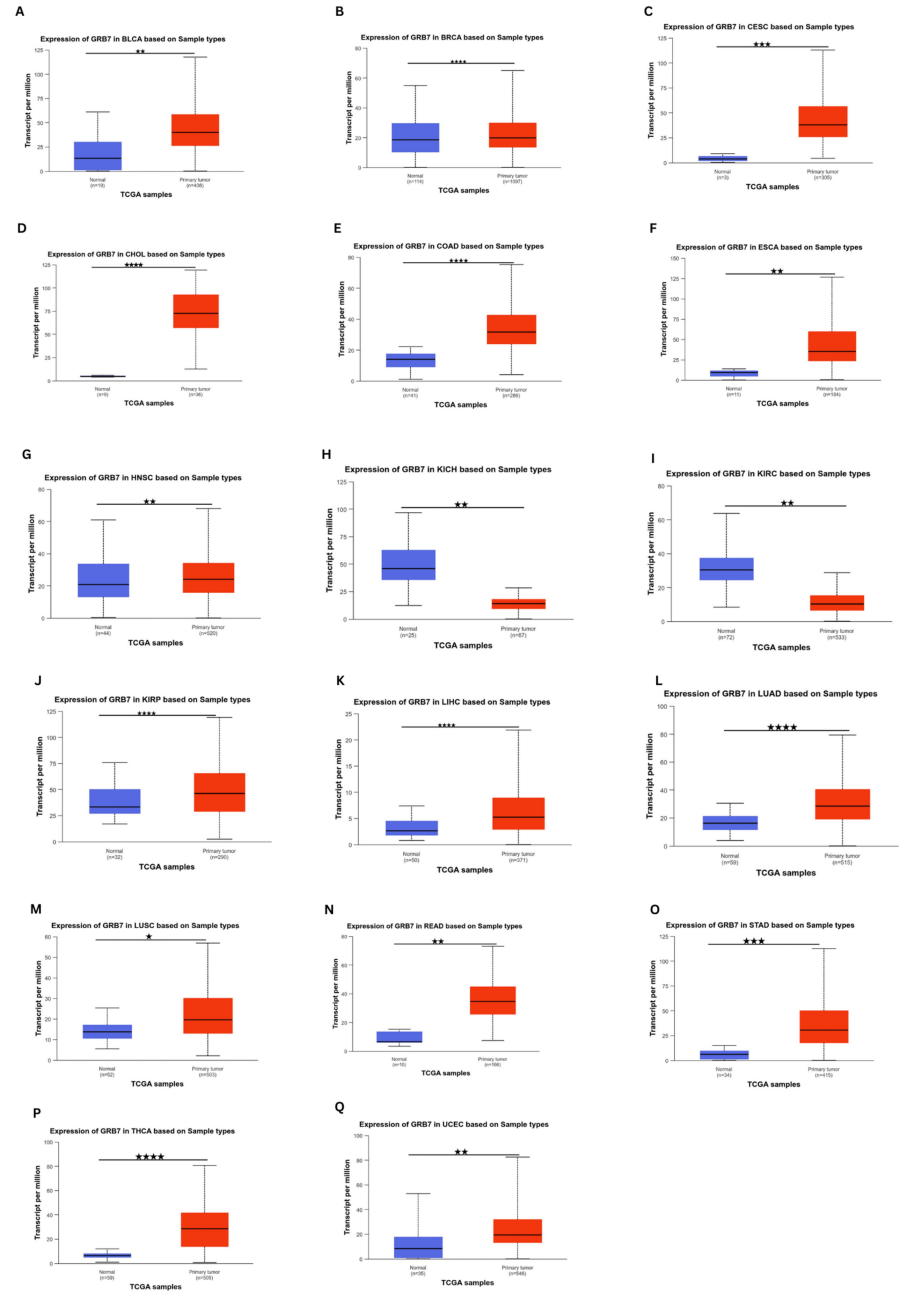
GRB7 expression levels in the tumor and normal tissues using UALCAN. The graphs showing the expression of GRB7 in (A) BLCA, (B) BRCA, (C) CESC, (D) CHOL, (E) COAD, (F) ESCA, (G) HNSC, (H) KICH, (I) KIRC, (J) KRIP, (K) LICH, (L) LUAD, (M) LUSC, (N) READ, (O) STAD, (P) THCA, and (Q) UCEC. The red columns represent the tumor tissues and the blue ones represent the normal tissues, while the stars indicate the differential significance between the tumor and normal samples. *P<1×10^-5^ was considered significant. **P<1×10^-9^ was considered significant. ***P<1×10^-11^ was considered significant. ****P<1×10^-13^ was considered significant. GRB7: growth factor receptor-bound protein 7; UALCAN: University of Alabama at Birmingham Cancer Data Analysis Portal; BLCA: bladder urothelial carcinoma; BRCA: breast invasive carcinoma; CESC: cervical squamous cell carcinoma and endocervical adenocarcinoma; CHOL: cholangiocarcinoma; COAD: colon adenocarcinoma; ESCA: esophageal carcinoma; HNSC: head and neck squamous cell carcinoma; KICH: kidney chromophobe; KIRC: kidney renal clear cell carcinoma; KIRP: kidney renal papillary cell carcinoma; LIHC: liver hepatocellular carcinoma; LUAD: lung adenocarcinoma; LUSC: lung squamous cell carcinoma; READ: rectum adenocarcinoma; STAD: stomach adenocarcinoma; THCA: thyroid carcinoma; UCEC: uterine corpus endometrial carcinoma

Due to the lack of normal controls for some tumors in the prior databases, a supplemental analysis was performed using the UALCAN database (Figures 2A-2Q). It indicated that GRB7 expression was significantly elevated in BRCA, CHOL, COAD, KIRP, LIHC, LUAD, and THCA (p<1×10^-13^), CESC, STAD (p<1×10^-11^), BLCA, ESCA, head and neck squamous cell carcinoma (HNSC), READ, UCEC (p<1×10^-9^), and LUSC (p<1×10^-5^). Conversely, GRB7 was significantly downregulated in KIRC and KICH (p<1×10^-9^). Whereas the expression of GRB7 in adrenocortical carcinoma (ACC), lymphoid neoplasm diffuse large B cell lymphoma (DLBC), mesothelioma (MESO), pancreatic adenocarcinoma (PAAD), and uveal melanoma (UVM) was found to be insignificant in all three databases.

**Figure 3:**
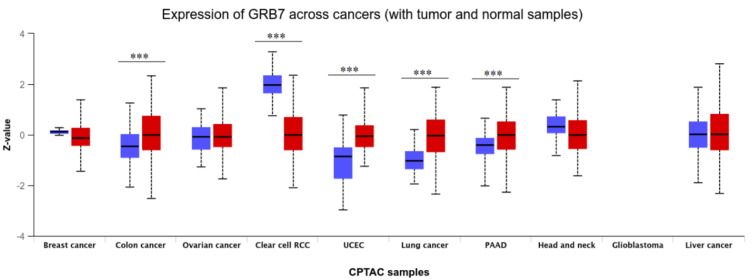
Pan-cancer analysis showing total protein expression of GRB7 using UALCAN. ***P<0.001 was considered significant. The red columns represent the tumor tissues and the blue ones represent the normal tissues while the stars indicate the differential significance between the tumor and normal samples. GRB7: growth factor receptor-bound protein 7; UALCAN: University of Alabama at Birmingham Cancer Data Analysis Portal; CPTAC: Clinical Proteomic Tumor Analysis Consortium; PAAD: pancreatic adenocarcinoma; UCEC: uterine corpus endometrial carcinoma

Moreover, the proteomic expression profiles of the GRB7 gene across various tumors were assessed using the UALCAN database. It was observed that GRB7 proteomic expression showed a significant upregulation in COAD, PAAD, lung cancer, and UCEC samples, while a decrease was noted in KIRC (p<0.001) (Figure 3). By cross-referencing the three databases (GEPIA, TIMER, and UALCAN), GRB7 expression was found to be significantly higher in BLCA, CESC, CHOL, COAD, READ, THCA, and UCEC. Conversely, it was significantly downregulated in KICH and KIRC.

Correlation between clinicopathological parameters and GRB7 expression in KICH and KIRC

**Figure 4:**
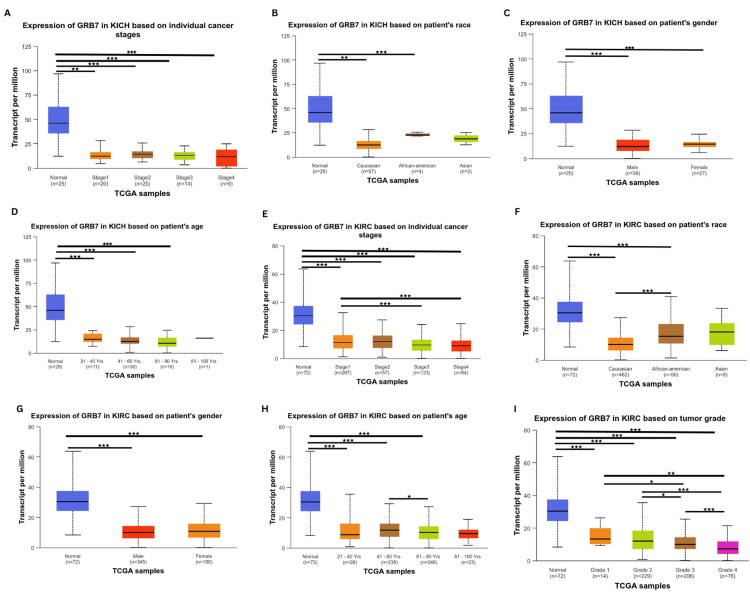
Correlation between clinicopathological parameters (stage, age, gender, race, and grade) and GRB7 expression in KICH and KIRC using UALCAN. Expression of GRB7 in KICH based on (A) stage, (B) race, (C) gender, and (D) age. Expression of GRB7 in KIRC based on (E) stage, (F) race, (G) gender, (H) age, and (I) grade. *P<0.05 was considered significant. **P<0.001 was considered significant. ***P<0.0001 was considered significant. GRB7: growth factor receptor-bound protein 7; UALCAN: University of Alabama at Birmingham Cancer Data Analysis Portal; KICH: kidney chromophobe; KIRC: kidney renal clear cell carcinoma; n: number of samples

**Figure 5:**
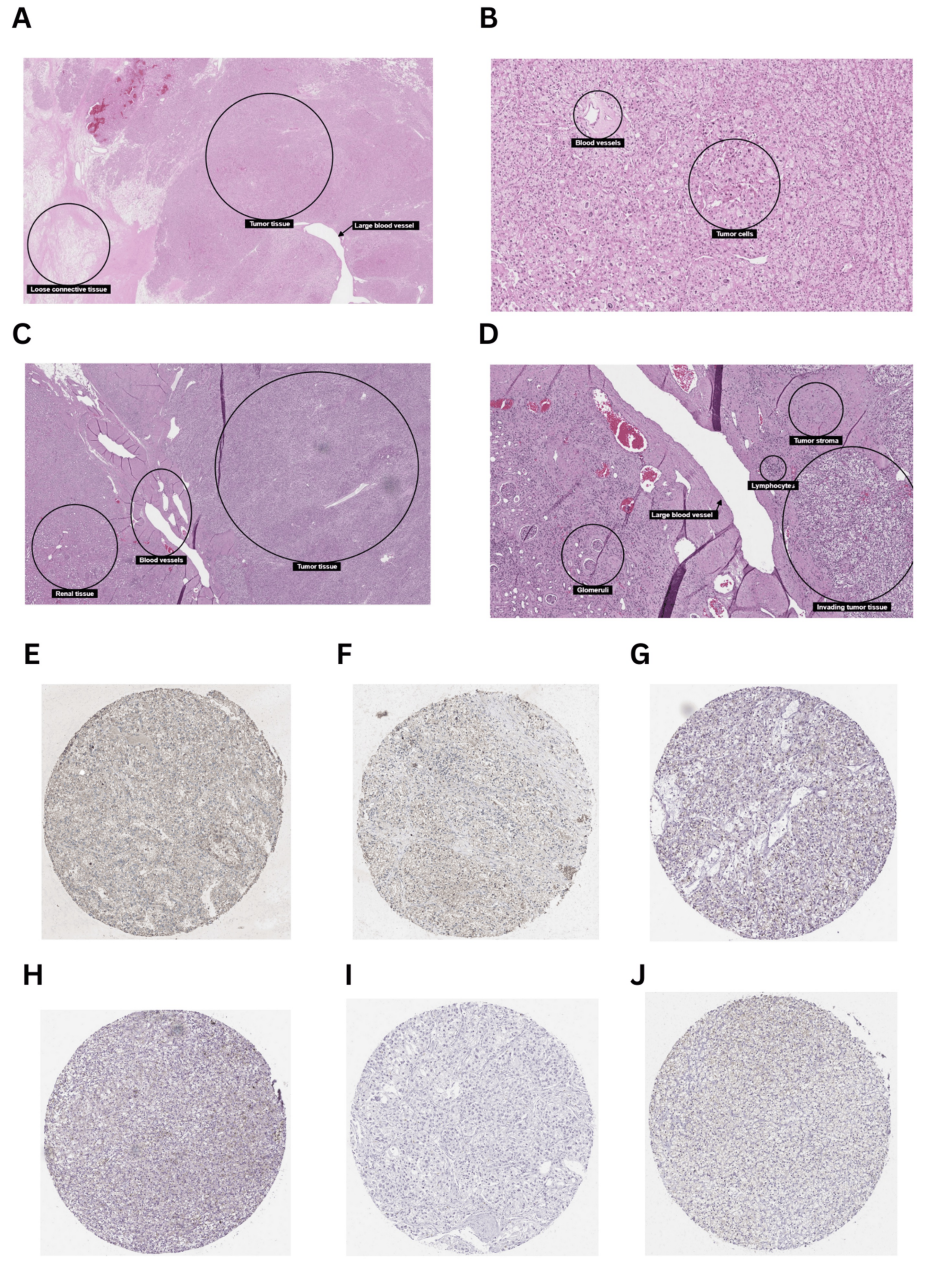
Immunohistochemistry of kidney cancer tissues using the Human Protein Atlas. (A) KICH tissue. (B) KICH tumor elements. (C) KIRC tissue. (D) KIRC tumor elements. (E) Kidney cancer tissue of a 52-year-old patient using antibody CAB005226. (F) Kidney cancer tissue of a 68-year-old patient using antibody CAB005226. (G) Kidney cancer tissue of a 59-year-old patient using antibody CAB073538. (H) Kidney cancer tissue of a 70-year-old patient using antibody CAB073538. (I) Kidney cancer tissue of a 59-year-old patient using antibody HPA057084. (J) Kidney cancer tissue of a 64-year-old patient using antibody HPA057084.

Considering the results obtained from the above analysis, KICH and KIRC were selected for further analysis using the UALCAN database. The correlation between clinicopathological parameters (stage, race, gender, and age) and GRB7 expression in KICH was investigated. The gene expression was significantly downregulated in stage 1 (p=1.66×10^-8^), stage 2 (p=4.52×10^-8^), stage 3 (p=6.81×10^-9^), and stage 4 (p=2.55×10^-4^) (Figure 4A). Furthermore, the gene expression profile showed a clear significance based on race (Figure 4B), with the gene GRB7 downregulated in Caucasians (p=1.50×10^-8^), and African Americans (p=9.92×10^-3^). Additionally, the gene expression profile showed high significance in the patient's gender (Figure 4C), with the gene GRB7 expressed in males and females, (p=1.38×10^-8^, p=2.77×10^-8^, respectively). Moreover, GRB7 was significantly downregulated in age groups 21-40 years (p=3.79×10^-8^), 41-60 years (p=2.12×10^-8^), and 61-80 years (p=8.12×10^-9^), as shown in Figure 4D. A detailed analysis of KIRC using the UALCAN database revealed significant alterations in gene expression across various clinicopathological parameters. Gene expression was consistently downregulated in all stages of KIRC compared to normal tissue, with highly significant p-values observed in stage 1 (p=1.62×10^-12^), stage 2 (p=1.52×10^-11^), stage 3 (p<1×10^-12^), stage 4 (p=1.62×10^-12^). Additionally, notable differences were detected between specific stages, such as stage 1 versus stage 3 (p=2.23×10^-4^) and stage 1 versus stage 4 (p=9.22×10^-4^) (Figure 4E). When analyzing different racial groups, significant downregulation in gene expression was found in both Caucasians (p<1×10^-12^) and African Americans (p=1.19×10^-10^), while no significant difference was observed in Asians (p=7.04×10^-1^). Furthermore, a significant difference was noted between Caucasians and African Americans (p=6.43×10^-5^) (Figure 4F). Gender-specific findings revealed significant downregulation in both males (p=1.62×10^-12^) and females (p<1×10^-16^), though there was no substantial difference between genders (Figure 4G). The age-based analysis demonstrated that gene expression was significantly lower across all age groups compared to normal, particularly in those aged 21-40 years (p=2.31×10^-7^), 41-60 years (p=1.11×10^-16^), 61-80 years (p<1×10^-12^), and 81-100 years (p=1.11×10^-16^). Furthermore, a significant difference was noted between the 41-60 years and 61-80 years age groups (p=1.84×10^-2^) (Figure 4H). In terms of cancer grade, gene expression was significantly downregulated across all grades compared to normal, with p-values as follows: grade 1 (p=1.20×10^-5^), grade 2 (p=2.55×10^-16^), grade 3 (p=1.62×10^-12^), and grade 4 (p<1×10^-12^). Additionally, significant differences were observed between specific grades - grade 1 versus grade 3 (p=1.39×10^-2^), grade 1 versus grade 4 (p=5.12×10^-3^), grade 2 versus grade 3 (p=2.48×10^-2^), grade 2 versus grade 4 (p=1.85×10^-8^), and grade 3 versus grade 4 (p=9.69×10^-5^) (Figure 4I). Moreover, the histology of kidney cancer tissue has been explored using the Human Protein Atlas showing the tumor elements of KICH and KRIC (Figures 5A-5J). In addition, cancer tissues were also compared between 41-60 years and 61-80 years age groups of patients with kidney cancer (Figures 5E-5J).

Prognostic value of GRB7 in cancers

**Figure 6:**
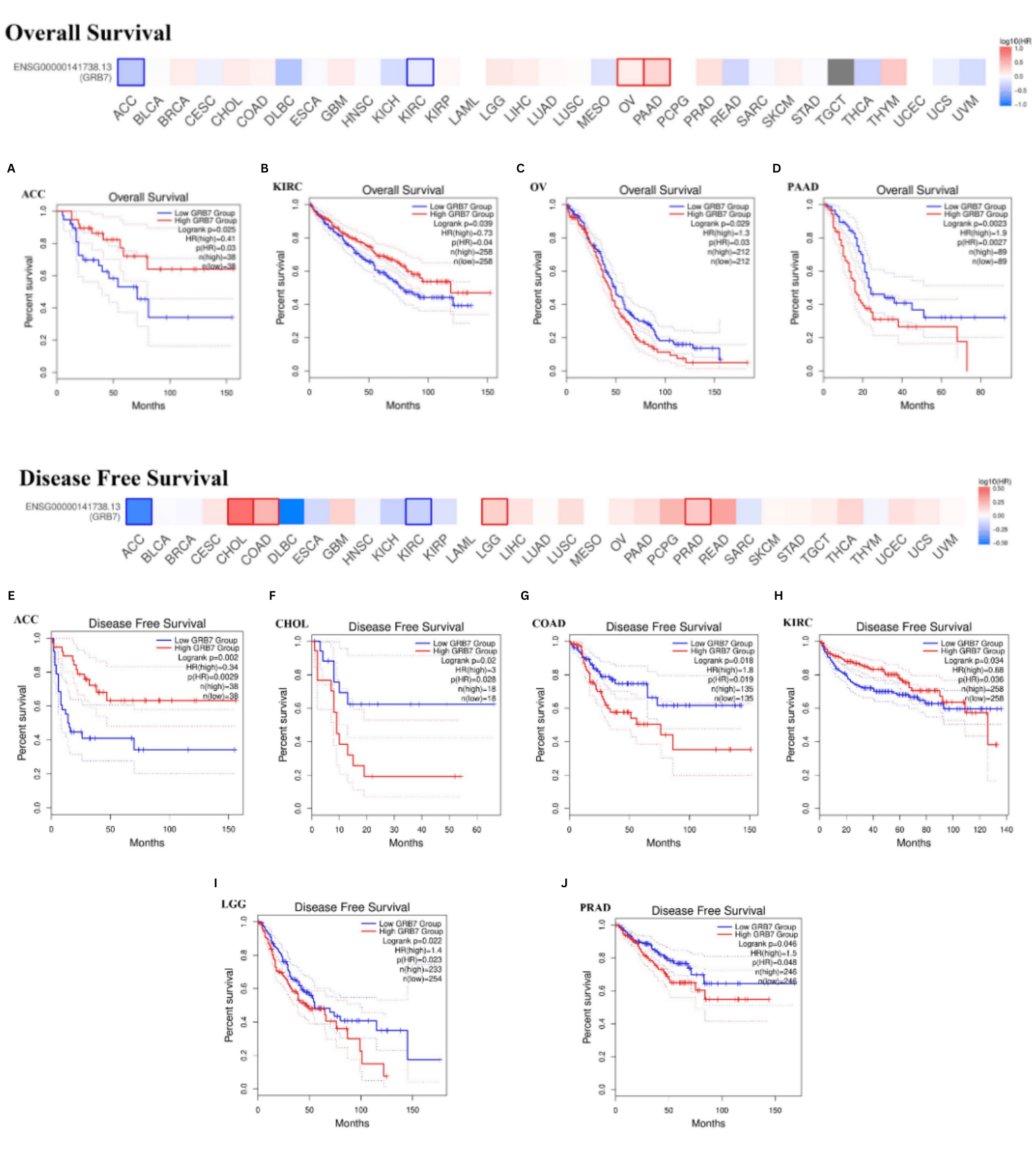
The correlation between GRB7 expression and the analysis of overall survival and disease-free survival in various cancers using GEPIA. The graphs show OS in patients with (A) ACC, (B) KIRC, (C) OV, (D) PAAD, and DFS in patients with (D) ACC, (F) CHOL, (G) COAD, (H) KIRC, (I) LGG, (J) PRAD. The red line represents high gene expression and the blue line represents low gene expression. P<0.05 was considered significant. GRB7: growth factor receptor-bound protein 7; GEPIA: Gene Expression Profiling Interactive Analysis; HR: hazard ratio; log-rank P: p-value resulting from log-rank test; OS: overall survival; DFS: disease-free survival; ACC: adrenocortical carcinoma; CHOL: cholangiocarcinoma; COAD: colon adenocarcinoma; KIRC: kidney renal clear cell carcinoma; LGG: brain lower grade glioma; OV: ovarian serous cystadenocarcinoma; PAAD: pancreatic adenocarcinoma; PRAD: prostate adenocarcinoma

Patients with cancer were categorized into groups with high and low GRB7 expression levels, and their overall outcomes were analyzed to evaluate the prognostic value of GRB7 (Figures 6A-6J). The GEPIA analysis indicated that higher GRB7 expression was linked with poorer overall survival (OS) in OV and PAAD (Figures 6C, 6D). However, increased GRB7 expression was correlated with better OS in patients with ACC and KIRC (p<0.05) (Figures 6A, 6B). Additionally, elevated GRB7 expression was related to poorer Disease-free survival (DFS) in patients with CHOL, COAD, LGG, and PRAD (Figures 6F, 6G, 6I, 6J). Meanwhile, higher GRB7 expression was associated with improved DFS in patients with ACC and KIRC (p<0.05) (Figure 6E).

**Figure 7:**
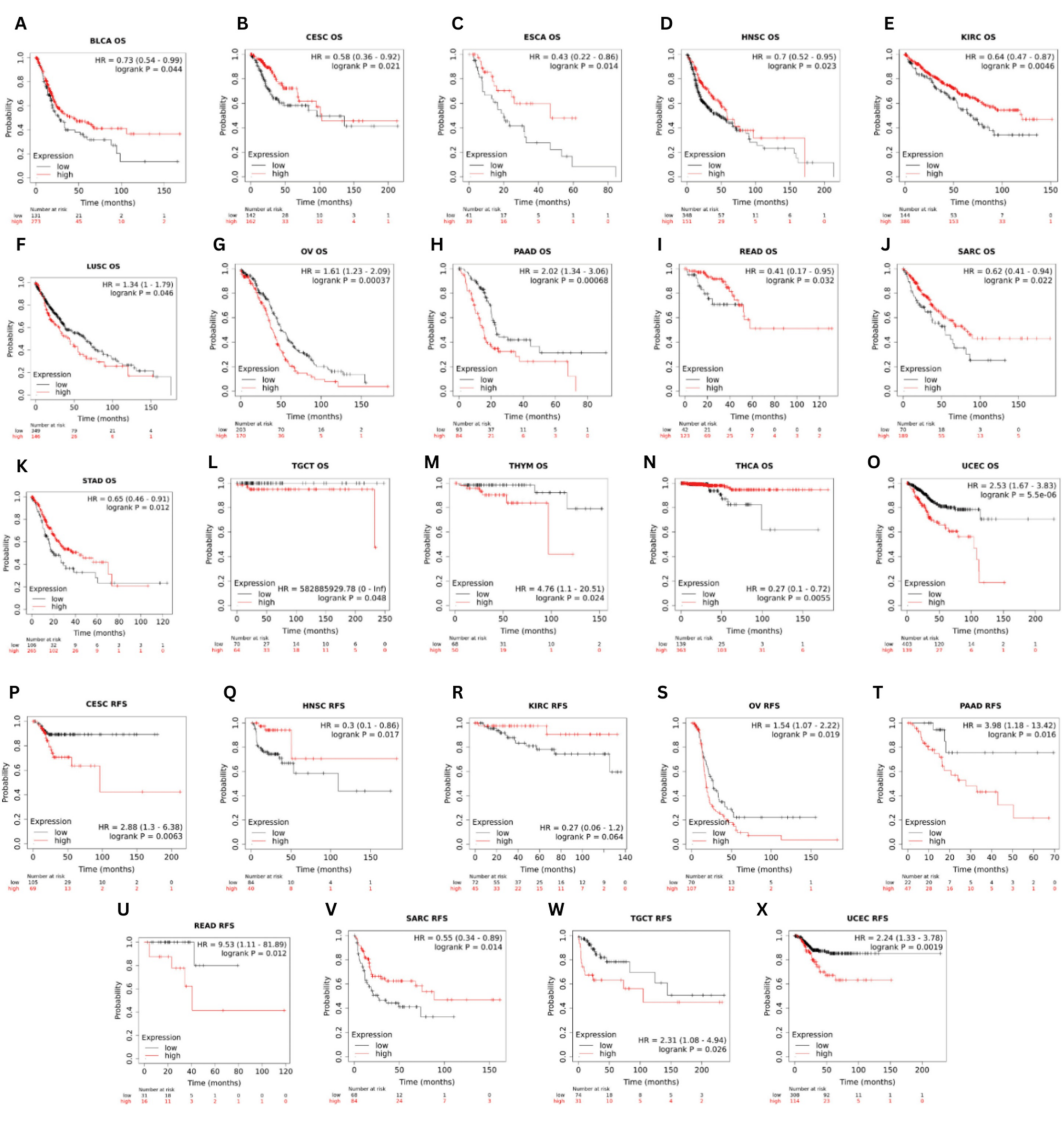
The correlation between GRB7 expression and the analysis of overall survival and relapse-free survival using Kaplan-Meier plotter. The graphs show OS in patients with (A) BLCA, (B) CESC, (C) ESCA, (D) HNSC, (E) KIRC, (F) LUSC, (G) OV, (H) PAAD, (I) READ, (J) SARC, (K) STAD, (L) TGCT, (M) THYM, (N) THCA, and (O) UCES, and RFS in patients with (P) CESC, (Q) HNSC, (R) KIRC, (S) OV, (T) PAAD, (U) READ, (V) SARC, (W) TGCT, (X) UCES. GRB7: growth factor receptor-bound protein 7; HR: hazard ratio, log-rank P: p-value resulting from log-rank test; OS: overall survival; RFS: relapse-free survival; BLCA: bladder urothelial carcinoma; CESC: cervical squamous cell carcinoma and endocervical adenocarcinoma; ESCA: esophageal carcinoma; HNSC: head and neck squamous cell carcinoma; KIRC: kidney renal clear cell carcinoma; LUSC: lung squamous cell carcinoma; OV: ovarian serous cystadenocarcinoma; PAAD: pancreatic adenocarcinoma; READ: rectum adenocarcinoma; SARC: sarcoma; STAD: stomach adenocarcinoma; TGCT: testicular germ cell tumors; THCA: thyroid carcinoma; THYM: thymoma; UCEC: uterine corpus endometrial carcinoma

The link between GRB7 expression and OS, or relapse-free survival (RFS), was explored using the Kaplan-Meier plotter database (Figures 7A-7X). The analysis showed that increased GRB7 expression was correlated with significantly poorer OS and RFS in OV (OS: HR=1.61, p=0.00037; RFS: HR=1.54, p=0.019), PAAD (OS: HR=2.02, p=0.00068; RFS: HR=3.98, p=0.016), TGCT (OS: HR=582885929.78, p=0.048; RFS: HR=2.31, p=0.026), and UCEC (OS: HR=2.53, p=5.5×10^-6^; RFS: HR=2.24, p=0.0019). In addition, patients with higher GRB7 expression had significantly poorer OS in LUSC (HR=0.73, p=0.044) and THYM (HR=4.76, p=0.024). However, significantly better OS and RFS were noted in HNSC (OS: HR=0.7, p=0.023; RFS: HR=0.3, p=0.017), and SARC (OS: HR=0.62, p=0.022; RFS: HR=0.55, p=0.014) for individuals with higher GRB7 expression. Furthermore, a significantly better OS was observed in BLCA (HR=0.73, p=0.044), ESCA (HR=0.43, p=0.014), KIRC (HR=0.64, p=0.00046), STAD (HR=0.65, p=0.012) and THCA (HR=0.27, p=0.0055). Whereas in CESC (OS: HR=0.58, p=0.021; RFS: HR=2.88, p=0.0063) and READ (OS: HR=0.41, p=0.032; RFS: HR=9.53, p=0.012), patients with higher GRB7 expression had better OS and poorer RFS.

**Figure 8:**
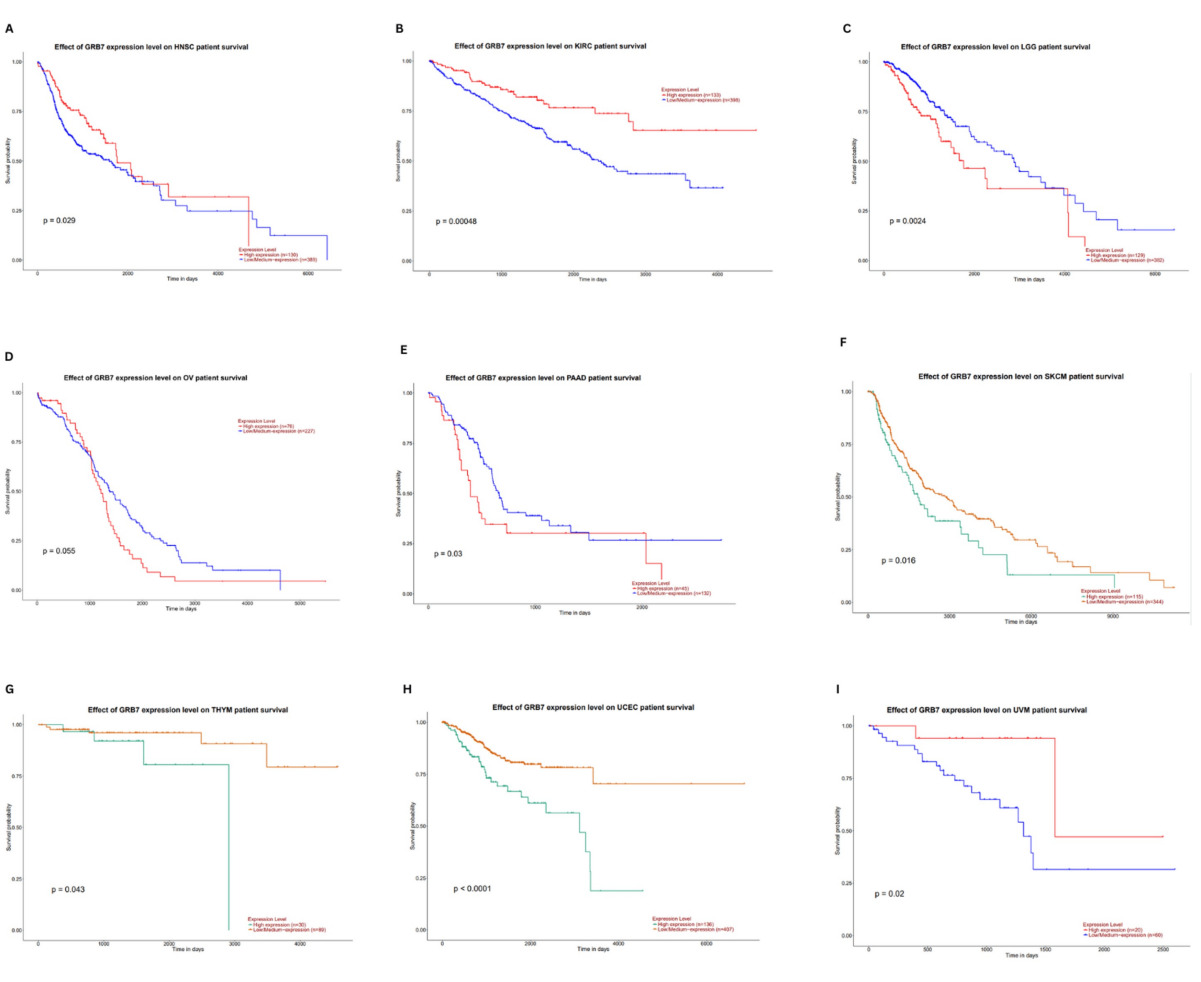
The analysis of overall survival based on the level of GRB7 gene expression in various cancers using UALCAN. The graphs show the effect of GRB7 gene expression on survival of patients with (A) HNSC, (B) KIRC, (C) LGG, (D) OV, (E) PAAD, (F) SKCM, (G) THYM, (H) UCEC, and (I) UVM. GRB7: growth factor receptor-bound protein 7; HR: hazard ratio; log-rank P: p-value resulting from log-rank test; TPM: transcripts per million; HNSC: head and neck squamous cell carcinoma; KIRC: kidney renal clear cell carcinoma; LGG: brain lower grade glioma; OV: ovarian serous cystadenocarcinoma; PAAD: pancreatic adenocarcinoma; SKCM: skin cutaneous melanoma; THYM: thymoma; UCEC: uterine corpus endometrial carcinoma; UVM: uveal melanoma

The relationship between GRB7 expression and overall survival (OS) was further examined using the UALCAN database. This analysis confirmed that higher GRB7 expression was linked with significantly better OS in HNSC (p=0.029), KIRC (p=0.00048), and UVM (p=0.02). The worst OS was noted in LGG (p=0.0024), PAAD (p=0.03), SKCM (p=0.016), THYM (p=0.043), and UCEC (p<0.0001) (Figures 8A-8I). Combining findings from GEPIA, TIMER, and UALCAN, high GRB7 expression was related to poor OS in PAAD, while it was linked to better OS in KIRC. This highlights the potential of GRB7 as a prognostic biomarker in these cancer types.

Correlation analysis of GRB7 expression and immune infiltration of immune cells

**Figure 9:**
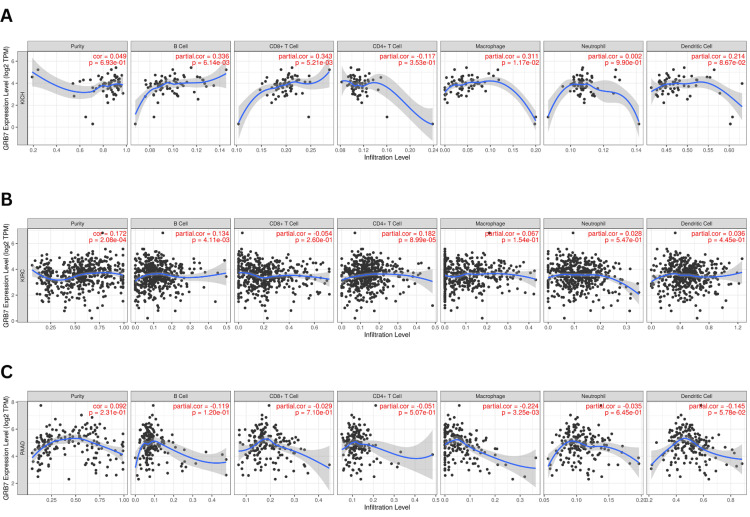
Correlation between GRB7 expression and immune infiltration of cells using the TIMER database. (A) KICH. (B) KIRC. (C) PAAD. GRB7: growth factor receptor-bound protein 7; TIMER: Tumor Immune Estimation Resource; KICH: kidney chromophobe; KIRC: kidney renal clear cell carcinoma; PAAD: pancreatic adenocarcinoma; B cells: B lymphocytes; CD8+ T cells: cytotoxic T lymphocytes; CD4+ T cells: T helper cells; Cor: correlation; log_2_ TPM: logarithm of 2 transcripts per million

The prognosis of tumors is influenced by the interaction of immune cells. In this study, the relationship between GRB7 expression in KICH, KIRC, and PAAD and the infiltration of immune cells, such as CD8+ T cells, CD4+ T cells, B cells, neutrophils, macrophages, and dendritic cells was investigated. In KICH, GRB7 expression was found to have a significantly positively correlated with B cell infiltration (partial. Cor=0.336, p=6.14×10^-3^), CD8+ cells (partial. Cor=0.343, p=5.21×10^-3^), and macrophage (partial. Cor=0.311, p=1.71×10^-3^), as shown in Figure 9A. Similarly, in KIRC (Figure 9B), GRB7 expression has significantly positively correlated with B cells (partial. Cor=0.134, p=4.11×10^-3^) and CD4+ cells (partial. Cor=0.182, p=8.99×10^-5^). While in PAAD a significant negative correlation between GRB7 expression and macrophage infiltration was observed (partial. Cor= -0.224, p=3.25×10^-3^), as shown in Figure 9C.

Genetic alteration analysis

**Figure 10:**
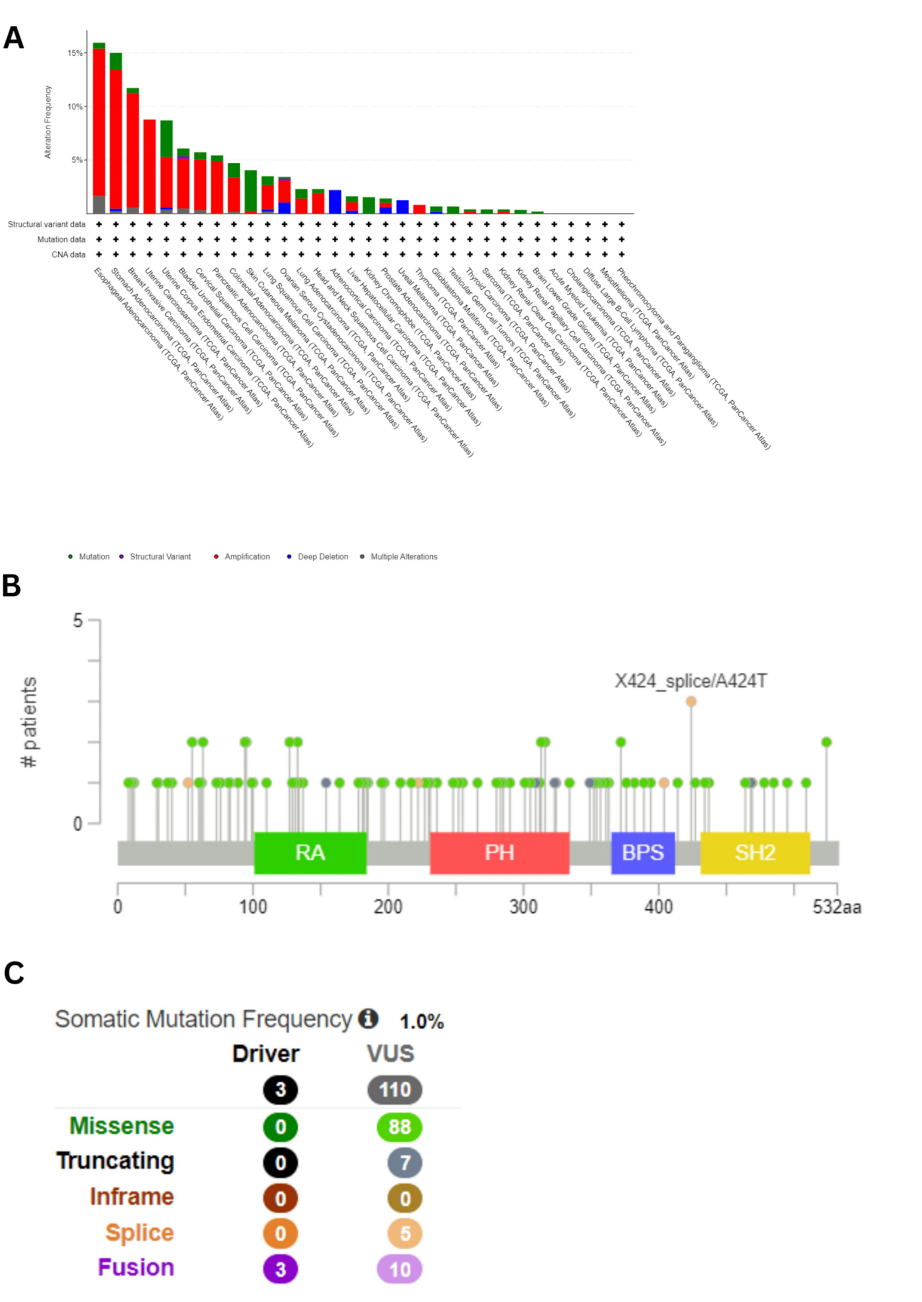
GRB7 genetic alterations analysis using cBioPortal platform. (A) GRB7 genetic alteration across frequency in 32 TCGA cancer types. (B) The sites and numbers of the GRB7 gene mutation types across protein domains in pan-cancer. (C) The number and types of GRB7 genetic mutations in pan-cancer. ESRG: embryonic stem cell-related gene; TCGA: The Cancer Genome Atlas; CNA: copy number alterations; log-rank P: p-value resulting from log-rank test

It is well-established that tumorigenesis is strongly linked with genetic alterations [7]. To examine the genetic alteration of GRB7 in human cancer, a comparative analysis was performed using the cBioPortal database. Among all 10,967 samples queried for 10,953 patients in 32 studies, GRB7 was found to be altered in 463 samples, accounting for approximately 4%. Amplification was identified as the most common type of genetic alteration in GRB7. For ESCA, STAD, BRCA, UCS, and UCEC, the highest frequencies of genetic alterations were observed, with rates of 15.93%, 15%, 11.72%, 8.77%, and 8.7%, respectively. Notably, mutations were more prevalent in SKCM, accounting for 3.83% (17/444 cases), and in UCEC, constituting 3.4% (18/529 cases), as shown in Figure 10A. In 10,967 samples, 113 mutation sites were identified between amino acids 0 and 532, including 88 missense mutations, seven truncating mutations, five splicing mutations, and 13 fusions, as shown in Figure 10C. The X424_splice/A424T mutation was found to be the most frequent. This mutation was situated between the BPS and the SH2 domain (Figure 10B).

Validation

**Figure 11:**
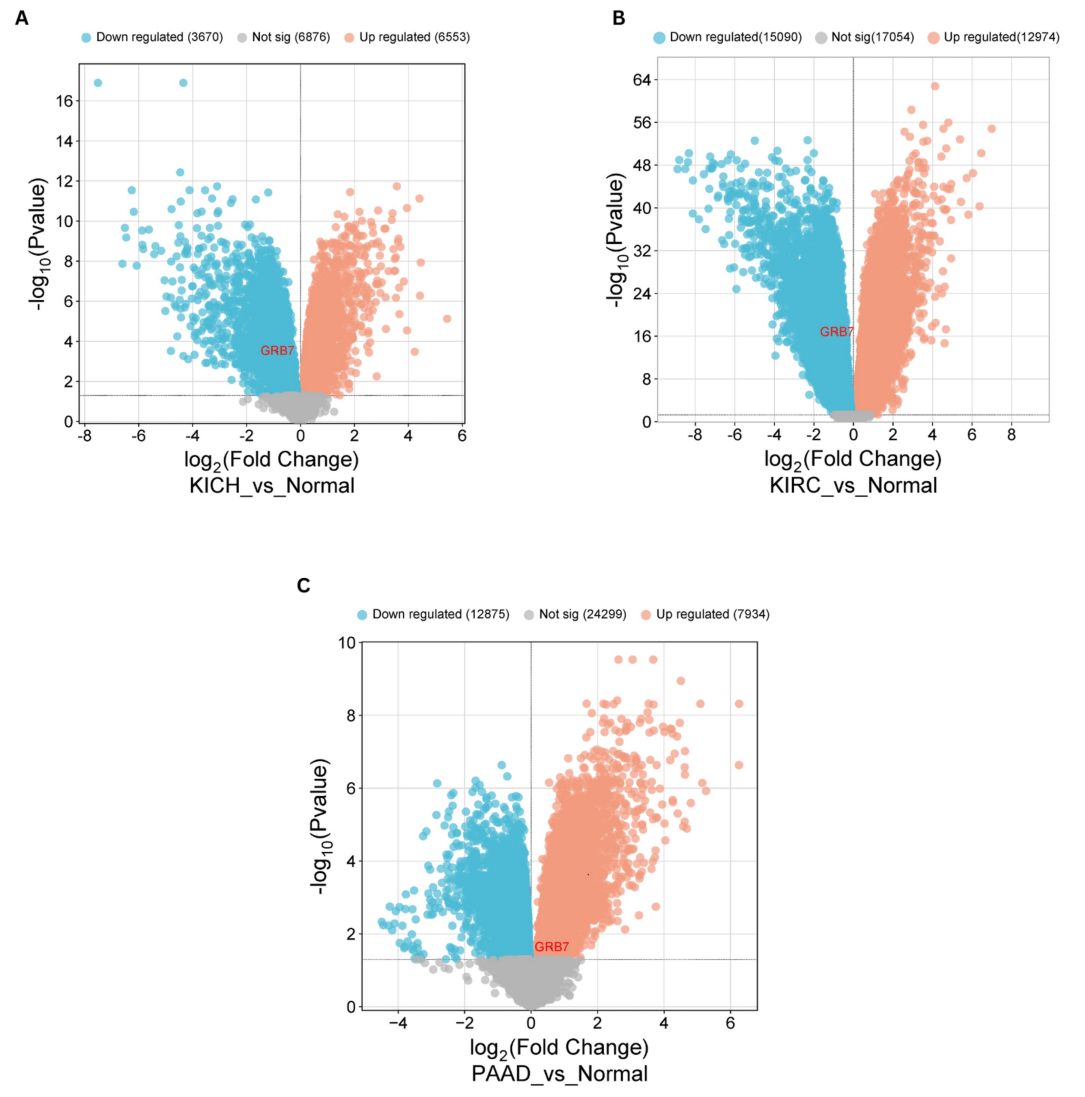
The volcano plot displays differential gene expression including the GRB7 gene in KICH, KIRC, and PAAD. (A) The validation data of GSE11024 dataset for KICH. (B) The validation data of GSE53757 dataset for KIRC. (C) The validation data of GSE16515 dataset for PAAD. Genes that are upregulated are shown as red dots, and those that are downregulated are shown as blue dots (|log_2_(FC)| > 1, adj. p<0.05). KICH: kidney chromophobe; KIRC: kidney renal clear cell carcinoma; PAAD: pancreatic adenocarcinoma The image is generated using the bioinformatics.com.

To validate our findings on GRB7 expression, gene expression profiling was performed using data from the Gene Expression Omnibus (GEO) database. A volcano plot was created with a bioinformatics tool (http://www.bioinformatics.com.cn) to visually represent the upregulation or downregulation of GRB7 among the differentially expressed genes (DEGs). In KICH, the study identified differentially expressed genes using the GEO2R tool, which compares sample groups to pinpoint significant differences in gene expression. The GSE11024 dataset was used, consisting of 12 normal samples in the control group and six tumor samples in the tumor group. GRB7 was significantly downregulated (adjusted p-value = 3.25×10^-4^; |log_2_(FC)| = -0.8512116). The analysis revealed 10,223 DEGs, of which 6,553 were upregulated and 3,670 were downregulated (Figure 11A). For KIRC, the GSE53757 dataset was used, the control group contained 72 normal samples, and the tumor group contained 72 samples. GRB7 was notably downregulated (adjusted p-value = 2.29×10^-17^; |log_2_(FC)| = -0.8176622). The analysis showed 28,064 DEGs, of which 12,974 were upregulated and 15,090 were downregulated (Figure 11B). In PAAD, the GSE16515 dataset was used, it contains 36 tumor samples and 16 normal samples. GRB7 was notably upregulated (adjusted p-value = 0.630; |log_2_(FC)| = 2.43×10^-2^). The analysis showed 20,809 DEGs, of which 7,934 were upregulated and 12,875 were downregulated (Figure 11C).

## Discussion

The identification of unique markers related to abnormal gene expressions is a critical approach for tumor diagnosis and treatment. GRB7 is located within the 17q12-21 amplicon in proximity to the HER2 gene [5], it plays a significant role in cellular signaling by interacting with various receptor tyrosine kinases and their downstream partners [4]. Consequently, GRB7 functions as a pivotal hub, linking diverse potential drivers of oncogenesis to downstream signaling pathways, thus presenting an appealing target for therapeutic intervention. However, a comprehensive bioinformatics analysis of GRB7's role across pan-cancer tissues using multiple databases has not yet been conducted.

This study examined the expression level of GRB7 in different malignancies using multiple databases, examining its association with gene expression, prognosis, immune infiltration, and gene alterations. The expression of the GRB7 gene was investigated simultaneously in three different databases. Statistically significant expression relative to normal tissue was observed in several cancers, with seven cancers (BLCA, CESC, CHOL, COAD, READ, THCA, and UCEC) showing significant upregulation across all databases. Conversely, GBR7 demonstrated a significant downregulation in expression levels in KICH and KIRC. These findings align with earlier research that identified GRB7 as being upregulated in various tumor types, such as cervical cancer [6], gastric cancer, bladder cancer, and hepatocellular carcinoma [17-19]. Further, the analysis in the UALCAN database confirmed significant downregulation of GRB7 expression in KICH across various clinicopathological parameters, including stage, race, and age, when compared to normal tissue. However, in KIRC, GRB7 expression showed significant variation, being lower in advanced stages compared to earlier stages, and also showed lower expression in Caucasians compared to African Americans when analyzing different racial groups. Additionally, older age groups show lower expression than younger age groups. A similar pattern was observed across cancer grades, as GRB7 expression decreased progressively with advancing grades. This difference in histology has been illustrated between kidney cancer tissues of patients aged 41-60 years and 61-80 years using antibodies CAB005226, CAB005226, and HPA057084. The crucial role of GRB7 in cancer prognosis was illustrated in many studies [17,18,20,21]. Although HER2 and GRB7 co-amplification was previously reported [22], another study done by Ramsey et al. suggested that GRB7 plays a unique role in oncogenic pathways independent of HER2, making it a potential standalone biomarker [23]. In this study, the significant role of GRB7 expression in prognosis was examined using multiple databases (Kaplan-Meier plotter, GEPIA, and UALCAN). Our results revealed that high GRB7 levels were connected to poorer OS in PAAD. This finding aligns with previous studies showing that increased expression of GRB7 protein is associated with regional lymph node metastasis in pancreatic cancer [24]. However, in KIRC, higher GRB7 expression is correlated with improved OS.

The tumor microenvironment (TME) plays a vital role in both tumor progression and the effectiveness of immunotherapy. It was found that the knockout of GRB7 was associated with increased T cell-mediated cytotoxicity, suggesting the possibility of GRB7 being a potential target for immunotherapy [25]. In this study, the relationship between GRB7 expression and immune cell infiltration in KICH, KIRC, and PAAD was examined using the TIMER database. For instance, in KICH, a significant positive correlation was observed between GRB7 expression and the infiltration of B cells, CD8+ T cells, and macrophages. While in KIRC, GRB7 expression was also positively correlated with the infiltration of B cells and CD4+ T cells. However, in PAAD, GRB7 expression showed a negative correlation with macrophage infiltration. These results indicate that GRB7 expression is linked to distinct patterns of immune cell infiltration within the TME, which may affect tumor progression and prognosis. Specifically, higher levels of GRB7 may promote the infiltration of B cells, CD8+ T cells, and macrophages while reducing macrophage presence in some cancers, suggesting that GRB7 plays a complex role in shaping the immune landscape across different cancers.

Significant findings regarding GRB7 were identified by analyzing genetic alteration data across various cancers using the cBioPortal platform. GRB7 exhibited genetic alterations in multiple cancer types, with amplification being the predominant alteration detected in ESCA, while mutations were notably prevalent in SKCM and UCEC. However, no significant impact of GRB7 mutations was observed in KICH, KIRC, and PAAD. Additionally, GRB7 gene expression in KICH, KIRC, and PAAD was validated using data from the GEO database and various online resources. The results showed that GRB7 expression was downregulated in KICH and KIRC, while it was upregulated in PAAD.

The primary limitation of the study is its dependence on computational datasets, making it essential to perform wet lab experiments to confirm these computationally derived results. Further experimental validation is needed to determine whether GRB7 is an independent adverse prognostic factor in PAAD and KIRC. In addition to exploring GRB7's role in modulating immune cell infiltration across the TME in KICH, KIRC, and PAAD to provide critical insights into its role in tumor progression and its potential as a target for immunotherapy-based strategies. These experimental studies will provide valuable insights into the potential of GRB7 as a therapeutic target.

## Conclusions

This study highlights that GRB7 was downregulated in KICH, suggesting its potential as a diagnostic biomarker for this cancer. Additionally, it could serve as a prognostic biomarker in PAAD and act as both a diagnostic and prognostic biomarker in KIRC. Moreover, GRB7 gene expression was observed to be associated with immune infiltration, highlighting its potential effect on the tumor microenvironment. The above results pinpoint the importance of GRB7 as a target for future research and therapeutic strategies in oncology.
